# Neuropathologic and molecular aspects of a canine distemper epizootic in red foxes in Germany

**DOI:** 10.1038/s41598-022-19023-9

**Published:** 2022-08-29

**Authors:** Franziska Geiselhardt, Martin Peters, Sven Kleinschmidt, Elisa Chludzinski, Melanie Stoff, Martin Ludlow, Andreas Beineke

**Affiliations:** 1 Department of Pathology, University of Veterinary Medicine Hannover, Foundation, Hanover, Germany; 2 Research Center for Emerging Infections and Zoonoses, University of Veterinary Medicine Hannover, Foundation, Hanover, Germany; 3 Chemisches und Veterinäruntersuchungsamt (CVUA) Westfalen, Arnsberg, Germany; 4 Lower Saxony State Office for Consumer Protection and Food Safety (LAVES), Food- and Veterinary Institute Braunschweig/Hannover, Brunswick, Germany

**Keywords:** Viral host response, Viral pathogenesis, Viral reservoirs, Virus-host interactions, Microbiology, Viral epidemiology, Immunopathogenesis, Infection, Inflammation

## Abstract

In the last fifteen years, an epidemic of canine distemper virus (CDV) with marked neurotropism has occurred in Europe after a longer period of endemic transmission. Many wildlife species have been infected, with red foxes (*Vulpes vulpes*) being particularly affected. Given that this species is assumed to mediate cross-species CDV infections to domestic and wild animals, tissue samples from foxes with confirmed CDV infection in North-Western Germany were investigated to better understand the neurotropic aspects of the disease. This analysis included histopathology, virus distribution and cell tropism, phenotyping of inflammatory responses and determination of the genotype of the viruses based on the phylogeny of the hemagglutinin (H) gene. The predominant lesion type is gliosis in both gray and white matter areas associated with an accumulation of Iba1^+^ macrophages/microglia and upregulation of major histocompatibility complex class II molecules in the brain, while sequestration of CD3^+^ T and Pax5^+^ B cell in CDV-infected foxes is limited. Demyelination is found in few foxes, characterized by reduced myelin staining with loss of CNPase^+^ oligodendrocytes in the cerebellar white matter and brainstem. In addition, axonal damage, characterized by β-amyloid precursor protein expression, is found mainly in these brain regions. In situ hybridization reveals a primary infection of the cerebral and cerebellar gray matter and brain stem. Iba1^+^ cells and NeuN^+^ neurons represent the main CDV targets. Sequencing of the CDV H open reading frame from fox tissues reveals that the virus strains belongs to three different sub-lineages of the Europe-1/South America-1 genotype, suggesting independent transmission lines.

## Introduction

Canine distemper virus (CDV) causes canine distemper in dogs and systemic and often fatal disease in multiple members of the order carnivora^1^. CDV is a member of the genus *Morbillivirus* in the family *Paramyxoviridae* and is most closely related to phocine distemper virus and the recently discovered porcine morbillivirus^2^ and Myotis bat morbillivirus^3^. CDV is an enveloped virus and contains a non-segmented, single-stranded negative-sense RNA genome, which encodes for six proteins: nucleocapsid (N), phospho- (P), matrix (M), fusion (F), hemagglutinin (H) and polymerase (L) proteins. Thus far, at least 20 genotypes of CDV have been identified, including the recently discovered Caspian and Asia 4–6 genotypes^4–8^.

Virus transmission is thought to happen via inhalation of aerosols or direct contact with infectious bodily fluids. Initial infection occurs in resident immune cells in the upper respiratory tract prior to spread to local lymph nodes from which the first viremic phase leads to systemic dissemination of CDV to lymphoid tissues, especially spleen and gut-associated lymphoid tissue^9^. A second viremic phase results in virus spread to epithelial cells of the respiratory, digestive, urinary and reproductive tract, as well as to resident cells of the central nervous system (CNS)^9^. Two cellular receptors for CDV have been identified, which are critical determinants of in vivo cellular tropism: CD150 (SLAMF1) is expressed by several immune cell types including activated B and T cells, dendritic cells and alveolar macrophages^10^, while nectin-4 (PVRL-4) is present at the adherent junctions of epithelial cells^11^. However, the mechanisms underlying virus spread to the CNS are not yet fully understood. It is known that CDV can enter the brain via infection of endothelial cells, infiltration of infected monocytes circulating in the blood and crossing the blood–brain barrier, as well as by them fusing with ependymal cells of the ventricles after entering the brain with the cerebrospinal fluid^12–15^. An additional route of CNS invasion occurs via anterograde axonal transport from nasal mucosal tissue through the cribriform plate to the olfactory bulb^15^.

CDV has a broad host range including members of the families *Canidae, Ursidae, Felidae, Procyonidae, Mustelidae, Ailuridae, Hyaenidae* and *Viverridae*^4^, as well as pinniped species from the families *Phocidae* and *Otariidae*^16–18^. Interestingly, severe distemper has also been documented in non-carnivore species such as marmots as well as rhesus and cynomolgus macaques^4,16,19^. This host promiscuity and the worldwide distribution of CDV poses a threat to several endangered species^17,18,20–22^. Several studies have previously reported CDV infection in different fox species^1,23–41^. Red foxes (*Vulpes vulpes*) are suspected to transmit the virus to other species due to their roaming behavior and increasing encroachment upon human settlements^27,42^. The seroprevalence of CDV in European foxes is estimated to be 36.8%^29,37,42^. In recent years, several outbreaks of distemper have been reported in Europe with an estimated mortality rate of 50%^29,31–33,37,39^. Large numbers of carnivores in Germany have either been found dead or shot due to abnormal behavior with associated neurological clinical signs. Pathological examination of carcasses has confirmed that CDV was the etiological agent responsible for this clinical syndrome.

Foxes infected with CDV exhibit similar clinical signs to those observed in dogs including respiratory disease, conjunctivitis, discharge from the airways and eyes, and severe diarrhea, leading to emaciation of the animal^23,27,32,35,38–40,43^. Occasionally, keratinous hyperplasia of the nose and footpads occurs^40^. Previous studies have shown that most infected animals present with neurological symptoms such as behavioral alterations^27,30–34,36,38,39,43^. These findings have been confirmed in farmed foxes in which additional symptoms are observed such as convulsions, ataxia, apathy and circling, as well as progressive paralysis and blindness^23,43^. Pathological examination of infected foxes often reveals a generally poor body condition with congested lungs, liver, and spleen^23,27,38,39,44^. Histologically, lesions consist of severe interstitial pneumonia exacerbated by secondary infections^23,27,35,36,38–40^. Lymphopenia due to lymphoid depletion becomes apparent in lymphatic organs, leading to generalized immunosuppression^27,35,38–40^.

A variety of lesions have been previously described in the CNS of foxes with a confirmed diagnosis of CDV infection^27,30,35–40^. The characteristics of CNS pathology in foxes is comparable to those of CDV-infected dogs^45–47^, but the relative prevalence of specific types of lesions appears to differ. For instance, gray matter involvement is more commonly seen in foxes compared to dogs following CDV infection ^46,48–50^. Factors determining the disease phenotype in dogs are age, immune status and the CDV strain^47,51,52^. However, more studies are required to better understand CDV infection of the fox CNS.

In the present study, a retrospective analysis of samples collected during an epizootic in red foxes in Germany with a particular focus on the neuropathology, phenotyping of cellular immune responses in the CNS and molecular characterization of the associated CDV strains was conducted.

## Results

The fox samples were collected from 2015 to 2018. Thirteen fox samples originated from districts in North-Rhine Westphalia, whereas five foxes were submitted for pathological investigation from three different districts in Lower Saxony. Eleven animals were of male sex, six of female sex and the sex of one animal was not reported. Carcasses of 14 adult, 2 juvenile and 2 foxes of unknown age were investigated. Necropsies of the fox carcasses revealed mainly interstitial to necrotizing pneumonia and bronchiolitis, lymphatic depletion in the spleen and intracytoplasmic and intranuclear inclusion bodies in the bladder epithelium. Organ samples, which were assessed histopathologically included brain, lung, spleen, bladder, kidney and liver of all animals. Of these organs, mainly the lung, spleen and bladder contained CDV^+^ cells detected by immunohistochemistry, as observed in CDV-infected dogs^1,67^. Postmortem examination revealed no evidence of concurrent infections with other pathogens in the CNS. A tabular overview of information on the foxes is given in supplemental table S1.

### Histopathology

**Figure 1:**
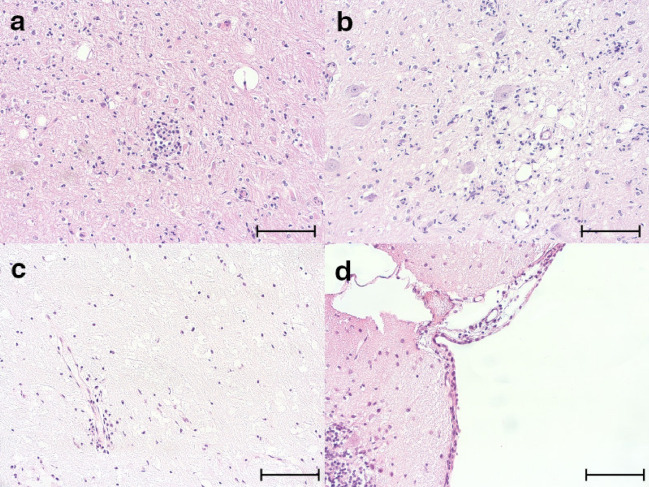
Histological lesions in fox brains. (**a**) Nodular gliosis, cerebellum. (**b**) Diffuse gliosis and vacuolization, cerebrum. (**c**) Mild perivascular lymphohistiocytic cuffing, cerebrum. (**d**) Meningeal lymphohistiocytic infiltrates, brainstem. (**a**–**d**) H&E staining. Scale bars = 100 µm.

Brain tissue of 18 foxes was analyzed for the presence of histopathologic lesions (Fig. 1a-d). The lesions were classified in the following categories: gliosis (nodular or diffuse accumulation of glial cells), perivascular inflammatory infiltrates, meningeal inflammatory infiltrates, vacuolization of brain matter, inclusion bodies, spheroids (swollen eosinophilic axons), and syncytia. Most animals showed several changes in the same brain region. The severity of pathologic changes varied in foxes, but most animals showed mild to moderate changes in all investigated brain regions. Yet, the hippocampal area seemed to be less affected as compared to the cerebral gray matter and brainstem. In all brain regions, nodular and diffuse glioses were the most prevalent lesion type, mainly found in the cerebral gray matter and brainstem (Table 1). Perivascular or leptomeningeal inflammatory infiltrates were also common.

**Table 1:** Distribution of histologic lesion types identified in infected foxes in different brain regions.

Brain region→ Lesion type	Cerebrum gray matter	Cerebrum white matter	Hippocampus	Cerebellum gray matter	Cerebellum white matter	Brainstem
Gliosis	15/17 (88%)	9/17 (53%)	6/17 (35%)	7/17 (41%)	11/17 (65%)	14/18 (82%)
Perivascular infiltration	9/17 (53%)	5/17 (29%)	0/17 (0%)	1/17 (6%)	6/17 (35%)	6/18 (33%)
Meningeal infiltration	9/17 (53%)	1/17 (6%)	1/17 (6%)	3/17 (18%)	0/17 (0%)	2/18 (11%)
Vacuolization	2/17 (5%)	0/17 (0%)	1/17 (6%)	0/17 (0%)	4/17 (24%)	3/18 (17%)
Inclusion bodies	3/17 (18%)	3/17 (18%)	0/17 (0%)	2/17 (12%)	1/17 (6%)	6/18 (33%)
Spheroids	0/17 (0%)	0/17 (0%)	0/17 (0%)	0/17 (0%)	0/17 (0%)	2/18 (11%)
Syncytia	0/17 (0%)	0/17 (0%)	0/17 (0%)	1/17 (6%)	0/17 (0%)	1/18 (6%)

Vacuolization was observed predominately in the cerebellar white matter (24% of animals) and brainstem (17% of animals), indicating myelin damage. Intranuclear and intracytoplasmic viral inclusion bodies were found in all brain regions except for the hippocampus, but mainly in the brainstem. Here, axonal damage with spheroid formation was also found. Syncytia formation was rare, with only one observation in the cerebellar gray matter and brain stem of one animal.

Overall, most histologic lesions were present in the cerebral gray matter, characteristic of polioencephalits, followed by the brainstem, cerebellar white matter, cerebral white matter, cerebellar gray matter and hippocampus. Histopathologic brain lesions of foxes are suggestive of an early disease phase, as observed in other wild carnivore species following CDV infection^39,113^.

### Viral antigen and RNA distribution and cell tropism

**Figure 2:**
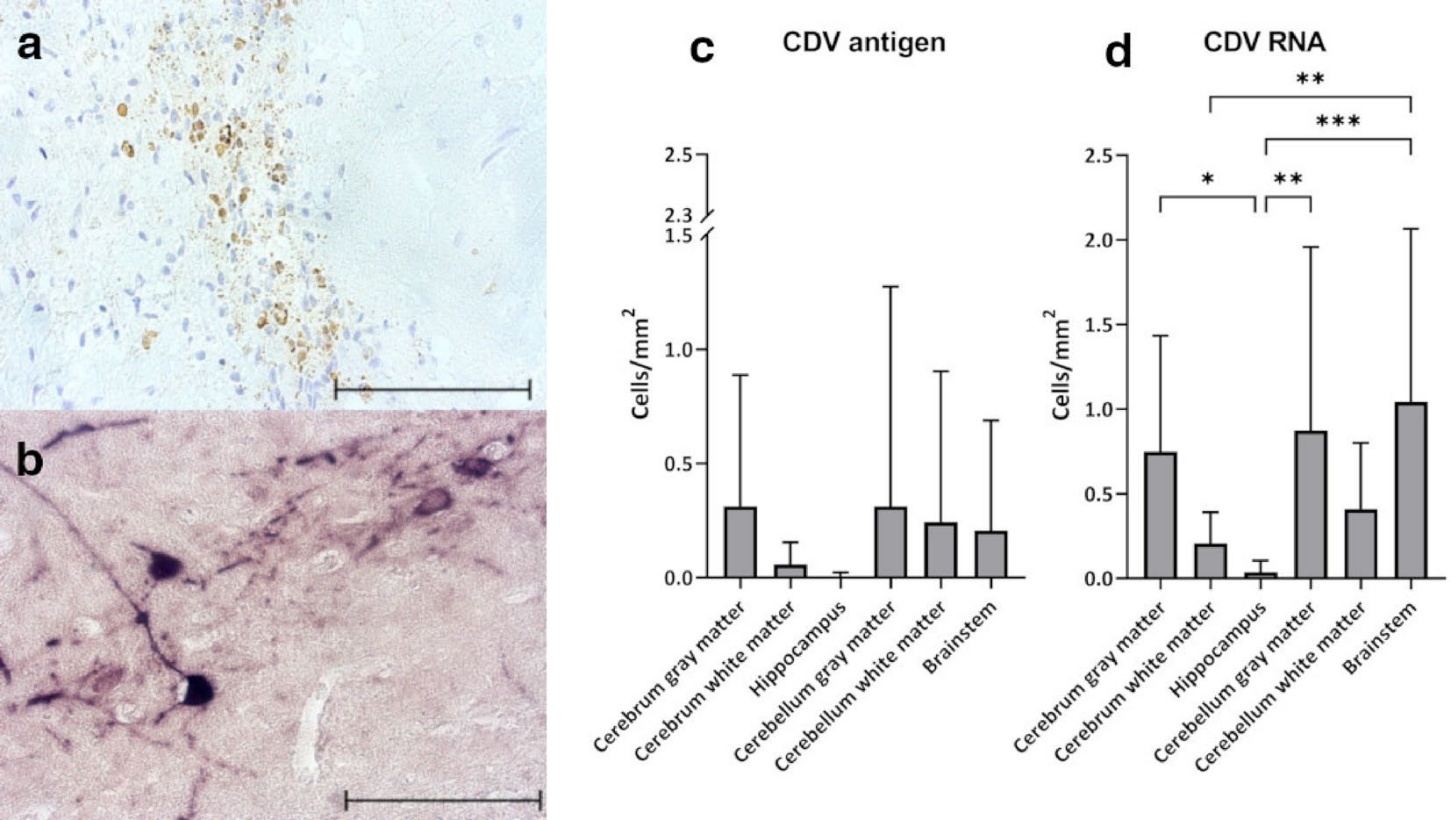
CDV antigen and RNA distribution in the brain of infected foxes. (**a**) CDV N antigen, immunohistochemistry, cerebellar white matter (**b**) CDV RNA, in situ hybridization, cerebrum. (**c**) Number of CDV N antigen-positive cells in different brain regions. (**d**) Number of CDV RNA-positive cells in different brain regions. (c, d) Bars display mean values and standard deviations. Asterisks indicate statistical significance levels, * ≤ 0.05, ** ≤ 0.01, *** ≤ 0.001. Scale bars = 100 µM.

The distribution of CDV antigen and RNA in brain regions was investigated by immunohistochemistry and in situ hybridization (ISH) (Fig. 2a–d). For each brain region, the number of animals, which showed CDV^+^ cells, was assessed. The regions where most animals were found to have CDV^+^ cells were the cerebral gray matter and the brainstem (Table 2). No significant changes were observed between brain areas by immunohistochemistry (Fig. 2c). However, lowest numbers of CDV nucleoprotein (N) antigen-positive cells were found in the hippocampus and cerebral white matter, whereas highest numbers of infected cells were identified in the cerebral and cerebellar gray matter. CDV^+^ foci in the brain matter were often located underneath meningeal areas (subpial). The leptomeninx of the cerebellum was found to harbor most CDV antigen as compared to the meninges of the brainstem and cerebrum.

**Table 2:** Numbers of animals, which showed positive staining for the indicated markers in the different brain areas.

Brain region staining	Cerebrum gray matter	Cerebrum white matter	Hippocampus	Cerebellum gray matter	Cerebellum white matter	Brainstem
CDV N protein	10/17 (59%)	7/17 (41%)	3/17 (18%)	6/17 (35%)	5/17 (29%)	10/18 (56%)
CDV N RNA	17/17 (100%)	17/17 (100%)	13/17 (76%)	17/17 (100%)	17/17 (100%)	17/18 (94%)
CNPase	2/17 (12%)	1/17 (6%)	1/17 (6%)	0/17 (0%)	3/17 (18%)	3/18 (17%)
LFB	1/17 (6%)	1/17 (6%)	1/17 (6%)	0/17 (0%)	3/17 (18%)	3/18 (17%)
β-APP	14/17 (82%)	10/17 (59%)	4/17 (24%)	2/17 (12%)	16/17 (94%)	16/18 (89%)

N: nucleoprotein, CNPase: 2,3′-cyclic–nucleotide 3′-phosphodiesterase, LFB: luxol fast blue, β-APP: beta-amyloid precursor protein.

The distribution of viral RNA in different brain areas was assessed by in situ hybridization. The overall numbers of CDV RNA-positive cells were higher than the number of cells stained by immunohistochemistry (Fig. 2c, d). The highest CDV RNA load was observed in the brainstem, followed by the cerebellar gray matter and cerebral gray matter (Fig. 2d). Although variance between samples was high, the number of CDV RNA-positive cells in the hippocampus was significantly lower compared to the brainstem, cerebellar gray matter and cerebral gray matter. Statistically significant differences were also found between the cerebral white matter and brainstem and cerebellar gray matter, respectively. Most animals showed CDV RNA in all brain regions, with the exception of four animals, which lacked positive staining in the hippocampus (Table 2).

**Figure 3:**
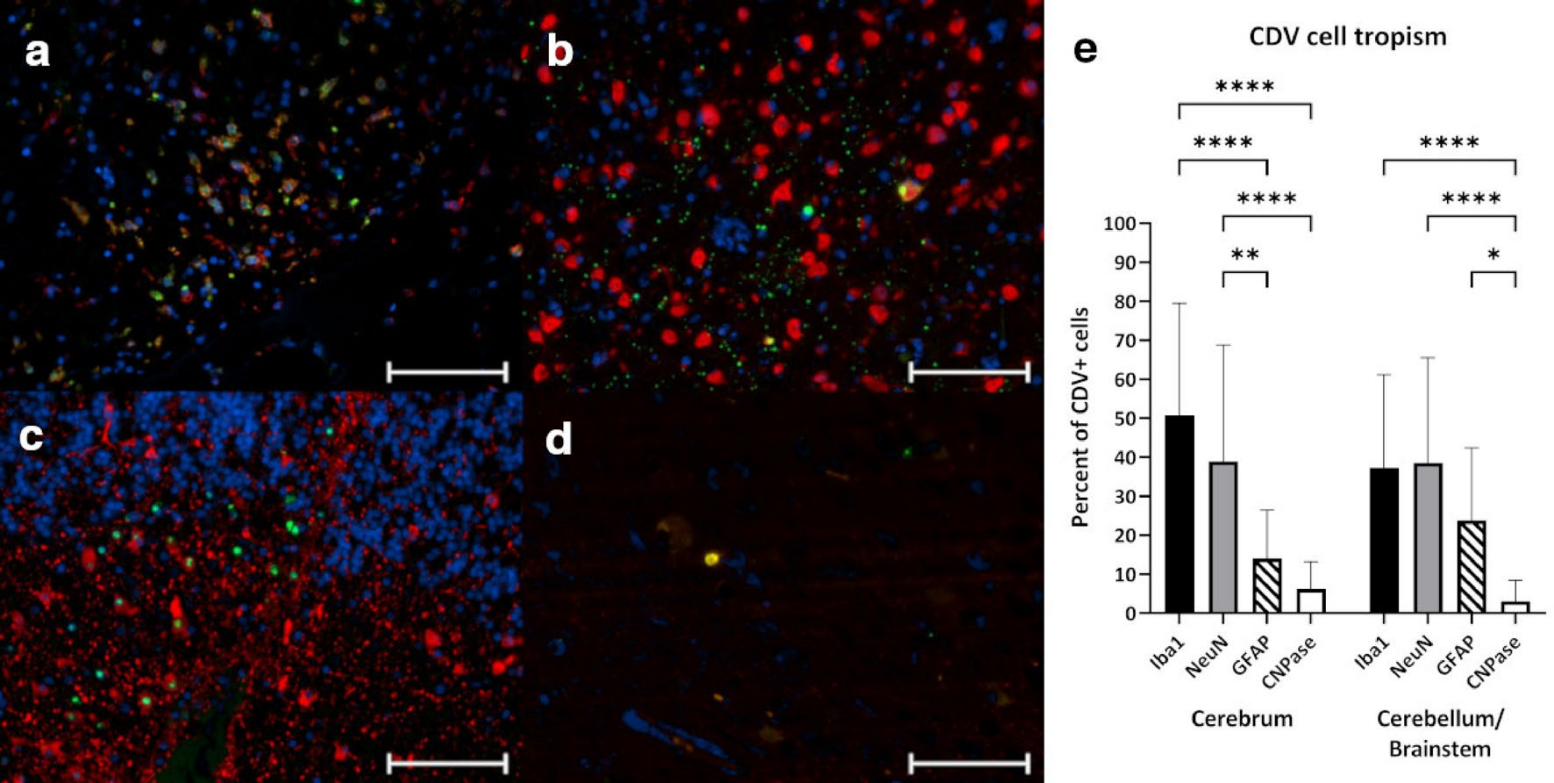
Cellular tropism of canine distemper virus in foxes. Detection of CDV N antigen (green) in (**a**) microglia/macrophages (Iba1, red), cerebrum. (**b**) neurons (NeuN, red), cerebrum. (**c**) astrocytes (GFAP, red), cerebellum. (**d**) oligodendrocyte (CNPase, red), cerebrum. (e) Percentage of infected cells displaying cell specific markers. Bars display mean values and standard deviations. Asterisks indicate statistical significance levels, * ≤ 0.05, ** ≤ 0.01, **** ≤ 0.0001 (**a**)–(**d**) immunofluorescence dual labeling, red: cellular marker, green: CDV N antigen, blue: nuclei. Scale bars = 100 µm.

To elucidate the viral cellular tropism, dual immunofluorescence stainings for the presence of CDV and markers glial acidic fibrillary protein (GFAP, astrocytes), neuronal nuclei (NeuN, neurons), ionized calcium binding adaptor molecule 1 (Iba1, microglia) and 2′,3′-cyclic-nucleotide 3′-phosphodiesterase (CNPase, oligodendrocytes), respectively, were performed (Fig. 3a–d). These cell types have been previously reported to be permissive to CDV infection^53–56^. Iba1^+^ microglia/macrophages were found to be the main CDV antigen-containing cell type with 50.8% of all CDV^+^ cells in the cerebrum and 37.3% CDV^+^ cells in the cerebellum (Fig. 3e). NeuN^+^ neurons comprised 38.9% of all CDV-infected cells in the cerebrum and 38.9% of CDV-infected cells in the cerebellum and brainstem. GFAP^+^ astrocytes represented 14.0% and 23.8% of infected cells, respectively. CDV antigen was observed only in single oligodendrocytes. In the cerebellum and brainstem, more CDV-infected neurons and astrocytes and less microglia/macrophages were observed as compared to the cerebrum. However, no statistical differences between the brain regions could be identified when comparing numbers of infected cells of a specific cell type.

### Cellular responses

**Figure 4:**
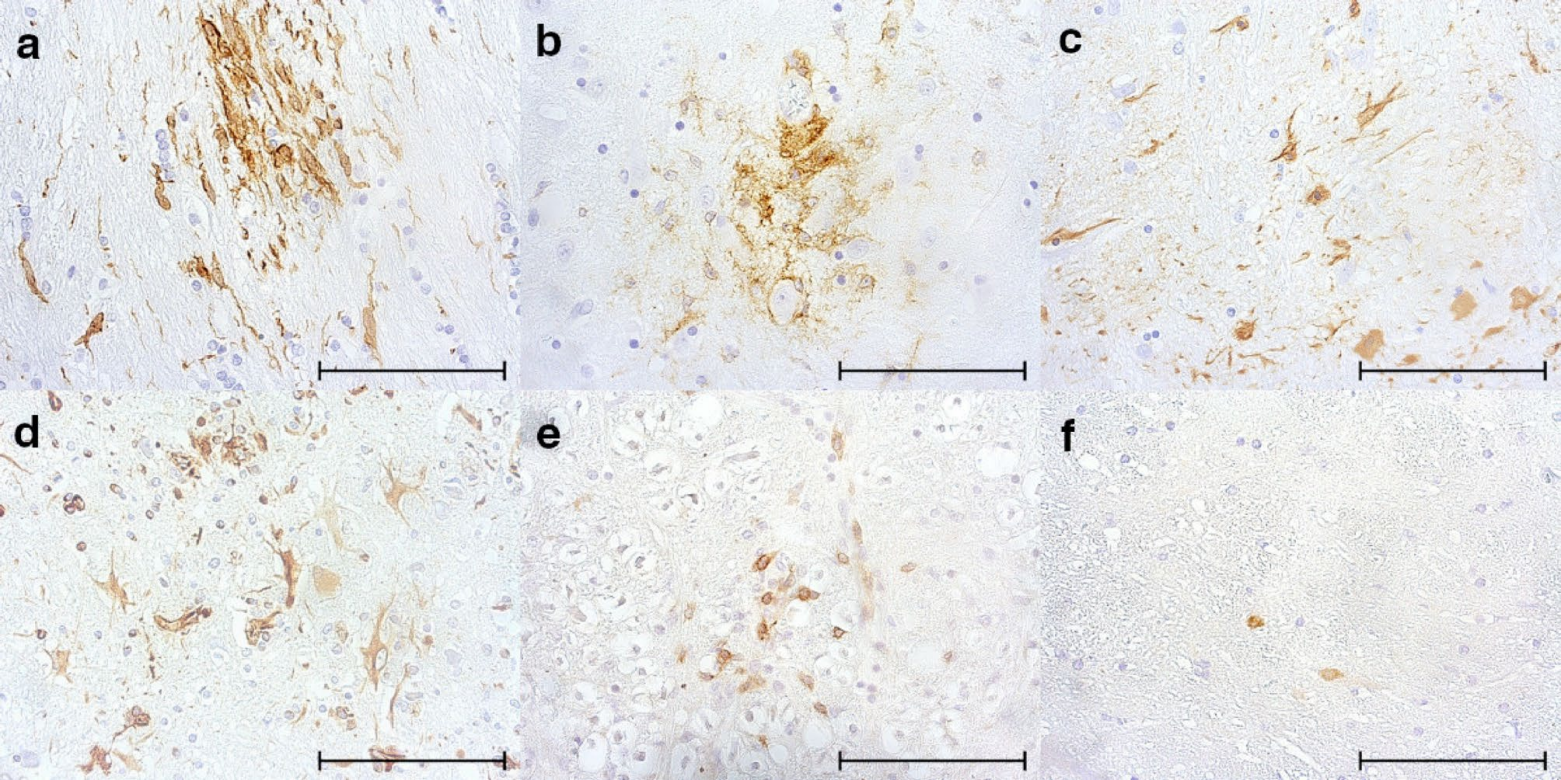
Phenotyping of cellular responses in the brain of infected foxes (**a**) Iba1^+^ microglia/macrophages, cerebrum. (**b**) MHC II expressing antigen-presenting cells, cerebrum. (**c**) GFAP^+^ astrocytes, cerebrum. (**d**) vimentin^+^ immature astrocytes, cerebrum. (**e**) CD3^+^ T cells, brainstem. (**f**) Pax5^+^ B cells, cerebrum. (**a**)–(**f**) immunohistochemistry, scale bars = 100 µm.

To further characterize CDV-induced pathology in fox brains, CNS resident and infiltrating immune cells were quantified by immunohistochemistry (Fig. 4a–f and Table 3). Accumulations of Iba1^+^ microglia/macrophages were found in all investigated brain areas, associated with an upregulation of MHC II. A significant upregulation of GFAP in the cerebral gray matter and brainstem, indicative of astrogliosis, was observed in CDV-infected animals compared to non-infected foxes. Expression of vimentin in astrocytic cells was generally low and variable within brain regions. Yet, no significant difference to control foxes was evident. However, vimentin^+^ cells appeared to be located in the center of astroglial nodules associated with loss of GFAP staining, indicating an accumulation of immature astrocytes, as found in brain lesions of CDV-infected dogs^103^. Evaluation revealed a mild but significant infiltration of CD3^+^ T cells in the cerebral gray matter, cerebral white matter, cerebellar gray matter, cerebellar white matter and brainstem of CDV-infected animals compared to non-infected foxes. No significant Pax5^+^ B cell infiltrates were found in any investigated brain region of infected foxes, probably related to virus-induced immunosuppression and depletion of peripheral lymphoid organs following infection^67^. In conclusion, phenotyping revealed predominantly innate immune responses and only limited adaptive immune responses in the CNS of foxes, characteristic of an acute to subacute CDV infection phase.

**Table 3:** Phenotyping of inflammatory responses in the brain of infected foxes.

Antigen			Cerebrum gray matter		Cerebrum white matter		Hippocampus		Cerebellum gray matter		Cerebellum white matter		Brainstem
Iba1	Median		2		2		2		2		2		2
	Max. score		3		3		3		2		3		2
	Significance		**		**		***		**		**		***
MHC II	Median		2		2		1		1		1		2
	Max. score		2		3		2		2		3		2
	Significance		**		**		***		*		ns		**
GFAP	Median		2		2		2		2		2		2
	Max. score		3		3		3		2		3		3
	Significance		*		ns		ns		ns		ns		*
Vimentin	Median		0		0		0		0		0		0
	Max. score		1		2		2		0		1		1
	Significance		ns		ns		ns		ns		ns		ns
CD3	Median		1		1		1		1		1		1
	Max. score		2		2		2		1		2		3
	Significance		***		***		ns		***		***		***
Pax5	Median		0		0		0		0		0		0
	Max. score		1		0		0		0		0		1
	Significance		ns		ns		ns		ns		ns		ns

0 = no observed immunoreactivity, 1 = single foci/cells, < 5%; 2 = multifocal reactivity, 5–30%; 3 = multifocal to diffuse reactivity, > 30%. Displayed in the table are median scores, and maximal scores observed in the respective areas, and the statistical significance levels as compared to uninfected control foxes. ns: not significant, * ≤ 0.05, ** ≤ 0.01, *** ≤ 0.001.

### Myelin and axonal damage

**Figure 5:**
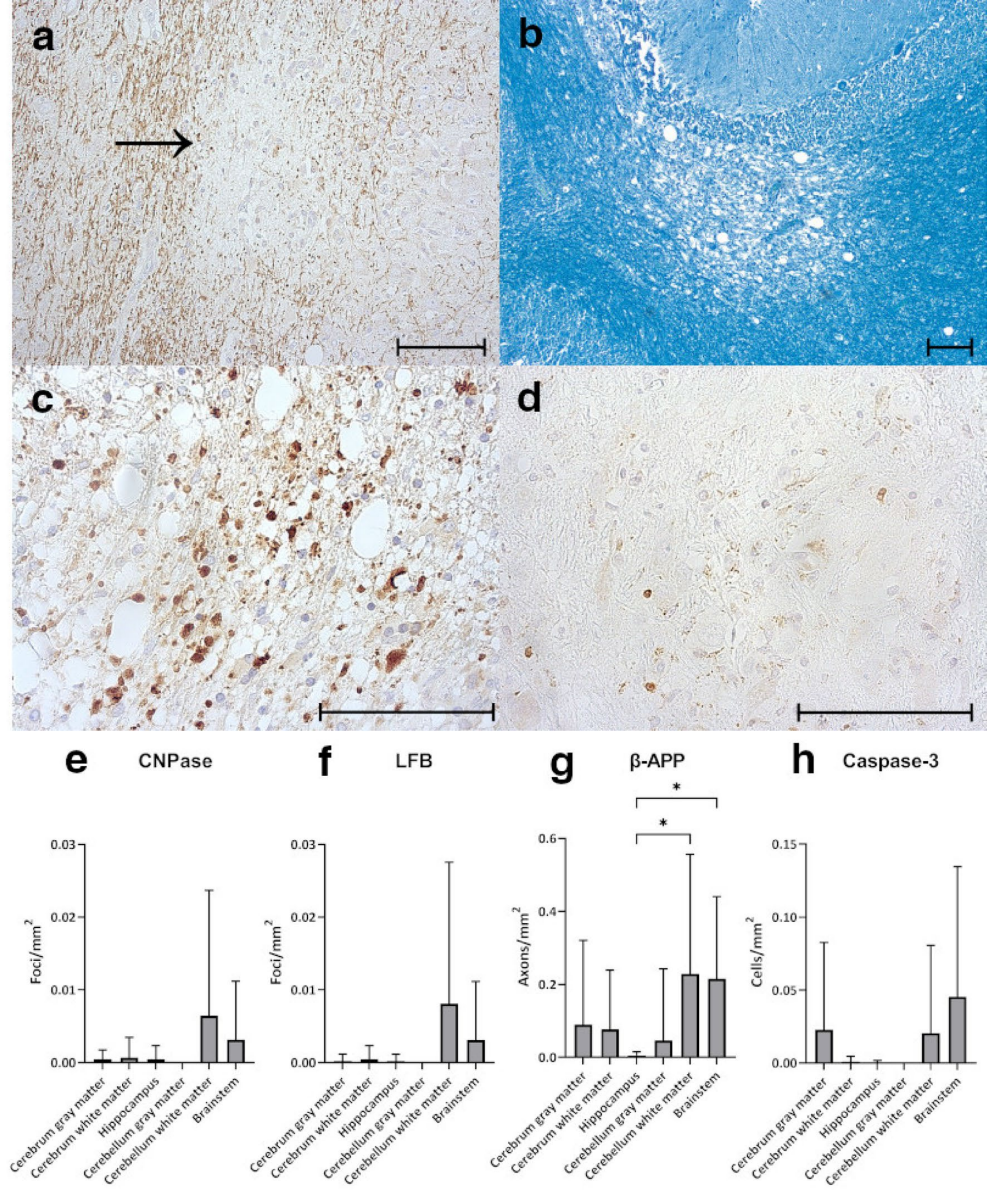
Identification of demyelination, axonal damage and apoptotic cells in infected foxes. (**a**) Lack of CNPase staining (arrow), cerebrum. (**b**) Lack of Luxol fast blue (LFB) staining, cerebellum. (**c**) β-APP^+^ damaged axons within demyelinating lesion, cerebellum. (**d**) Cleaved caspase-3^+^ apoptotic cells, cerebrum. (**e**) Quantification of foci lacking CNPase staining (CNPase-negative foci) within different brain regions. (**f**) Quantification of foci lacking LFB straining (LFB-negative foci) within different brain regions. (**g**) Quantification of β-APP^+^ axons within different brain regions. (**h**) Quantification of cleaved caspase-3^+^ apoptotic cells within different brain regions. (**e**–**h**) Bars display mean values and standard deviations. Asterisks indicate statistical significance levels, * ≤ 0.05. Scale bars = 100 µm.

A distinctive feature of CDV infection of dogs is the development of demyelinating disease and axonopathy^12,57^. In order to detect demyelination in the brain of CDV-infected foxes immunostaining for CNPase and luxol fast blue (LFB) staining have been performed (Fig. 5a, b, e, f). Loss of LFB myelin staining and CNPase^+^ oligodendrocytes was identified mainly in the cerebellar white matter and brainstem, associated with vacuolization of the neuroparenchyma. However, due to low numbers of demyelinating foci and variations between animals, no significant differences between brain regions were detected. Occasionally, myelinophages containing LFB^+^ material within the cytoplasm were detected within foci. To examine brain tissue for the presence of axonal damage, expression of beta-amyloid precursor protein (β-APP), a marker for axonal degeneration, has been analyzed by immunohistochemistry (Fig. 5c and g). β-APP^+^ damaged axons were present in all investigated brain regions, albeit at different numbers. Most damaged axons were found in the cerebellar white matter and brainstem, often located within demyelinating foci. Significantly lower numbers of β-APP^+^ damaged axons were found in the hippocampus. Again, numbers of positive axons differed markedly between investigated animals, leading to high standard deviations.

Apoptotic cells were determined by cleaved caspase-3-specific immunohistochemistry (Fig. 5d and h), given that CDV is known to cause apoptosis^58–60^. The majority of apoptotic cells were located in the brainstem, followed by the cerebral gray matter and cerebellar white matter. However, inter-sample differences were too high to detect statistical differences between brain regions. Few apoptotic cells were located in the cerebral white matter and hippocampus, whereas no apoptotic cells were detected in the cerebellar gray matter. Apoptotic cells were often located also within the meninges and perivascular spaces, suggestive of apoptotic immune cells, similar to findings in dogs with canine distemper^58,59^.

### Molecular characterization of CDV fox strains

**Figure 6:**
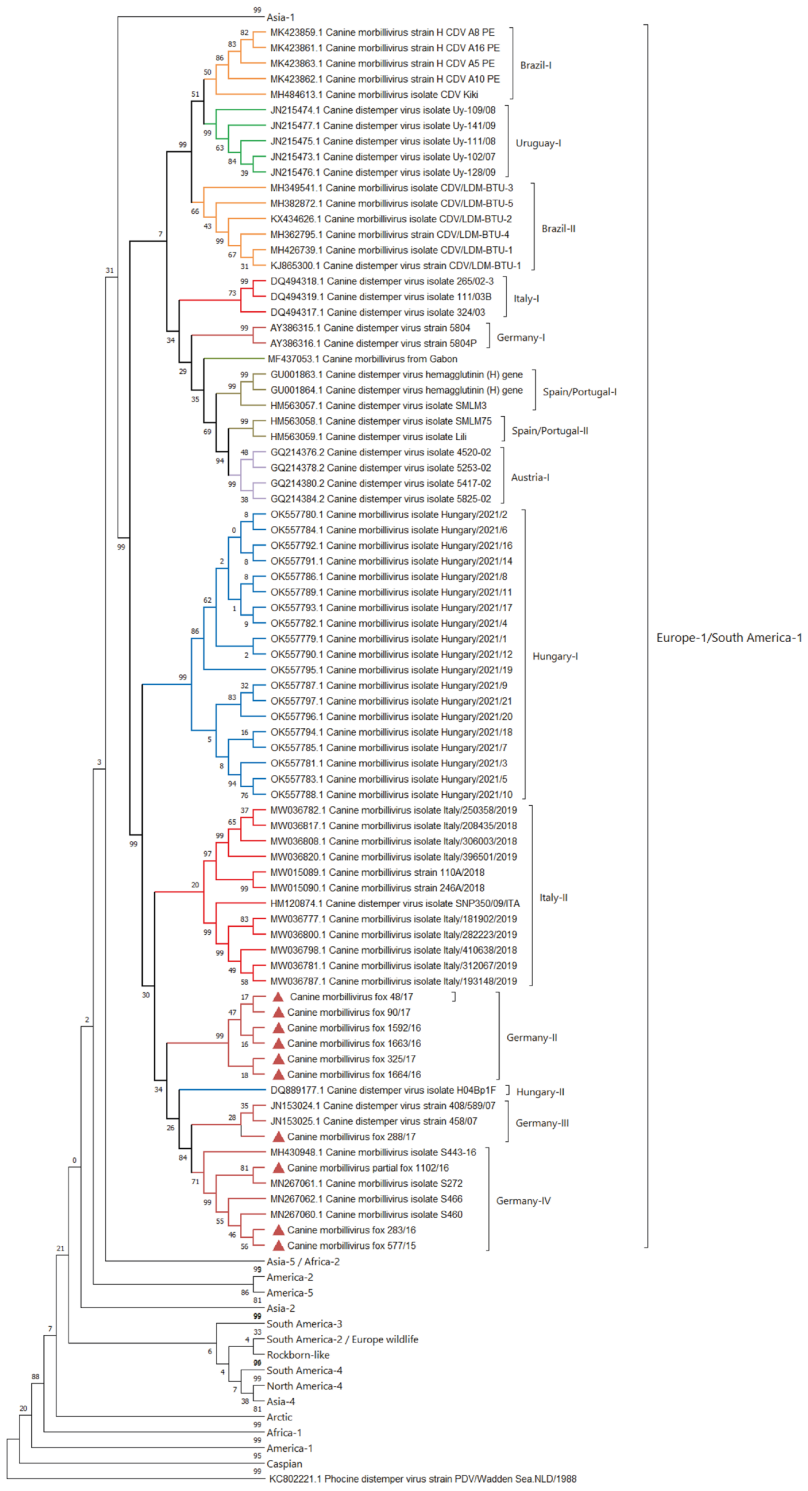
Maximum-likelihood phylogenetic tree of 600 complete CDV-H gene sequences including ten H gene sequences amplified from fox tissue. Bootstrap values are presented at nodes. The H gene sequence of phocine distemper virus (GenBank accession no. NC028249.1) was used as an outgroup. Foxes from this study are indicated by filled triangles (red) at tip ends. Numbers on the nodes indicate bootstrap values.

The evolutionary relationship of CDV strains present in fox tissues to strains previously detected in Germany was determined by amplifying the CDV H open reading frame (ORF) by RT-PCR from ten animals and Sanger sequencing of resulting amplicons. These sequences were aligned to 600 other complete CDV H ORF sequences available on GenBank (https://www.ncbi.nlm.nih.gov/genbank/). A maximum-likelihood phylogenetic tree was constructed to identify the genotype of the different CDV strains present in the fox samples under investigation (Fig. 6). All CDV strains could be classified within the Europe-1/South America-1 lineage but were divided into three closely related subclades (Germany-II, -III, -IV) which differed from an older CDV strain (5804) isolated in Hannover, 1989 (Germany-I). Six fox strains (48/17, 90/17, 1592/16, 1663/16, 325/17, 1664/16) formed a unique subclade which has been termed ‘Germany-II’. One fox strain (288/17) shares a common ancestor with strains that infected two German foxes in 2008 and strains present on a mink farm in Denmark in 2012^34,61^. This subclade was tentatively named ‘Germany-III’ and was related to a CDV strain from Hungary from 2004 which has been recently proposed to be the origin of the ongoing CDV epidemic in central Europe in the last fifteen years^62^. The final three fox viruses (1102-16, 283-16, 577-15) cluster closely with CDV strains (Germany-IV) originating in a fox, raccoon and a dog, which have recently been phenotypically characterized in a previous study, originating from the same area in North Rhine-Westphalia^5^. Further analysis was performed to identify unique amino acids present in the H protein of the fox CDV strains in comparison to representative related strains from the Germany-I, -II, -III and –IV subclades (Figure S2). Fox strains within the Germany-II subclade showed 100% homology on amino acid levels, whereas the four fox strains from the Germany-III and -IV subclades were more heterogeneous in their protein composition. The fox 288/17 strain (belonging to the Germany-III subclade) showed several individual amino acid mutations (F70I, L233V, E369G) including an additional N-linked glycosylation site (D584N) in comparison to H proteins from the other CDV-infected foxes. Furthermore, the fox CDV strains differed at position 549 in the H protein, which is involved in binding to the cellular receptor CD150^63,64^. Three strains contained a histidine residue at position 549, whereas the other seven strains contained a tyrosine residue. The other CD150 binding sites (e.g. aa 525-529 and 552) appeared to be conserved^65,66^. Although fox CDV strains were found to differ with respect to affiliation to specific subclades with the Europe-1/South American-1 lineage and showed some variation in the receptor-binding domain of the H protein, no differences in neuropathology between foxes could be linked to specific virus molecular determinants.

## Discussion

In this study, characteristics of CDV infection of foxes with special focus on the pathology and virus distribution in the brain were investigated. Most animals showed polioencephalitis with gliosis and infrequently demyelination in the white matter of the cerebellum and brainstem. Results indicate a rapid disease course as commonly found in wild canids and probably immunologically naïve hosts following CDV infection^51,113^.

Pathologic analysis of CDV-infected foxes showed that lesions were present in all brain regions, with nodular and diffuse gliosis found to be predominant. The most severe changes were observed in the brainstem and cerebral gray matter. Demyelinating plaques were found in only few animals mainly in the cerebellar white matter and brainstem, characterized by a loss of myelin staining and CNPase^+^ oligodendrocytes together with the presence of β-APP^+^ damaged axons. Similar to the present study, polioencephalitis with limited myelin damage was a main finding in wild carnivores, including foxes, badgers and martens, observed during a CDV epidemic in Switzerland^39,113^. Neuropathology in CDV-infected foxes is indicative of an acute to subacute disease course, because, firstly, the observation that the cerebral gray matter was heavily affected corresponds to early lesion development in CDV-infected dogs^9,67^. Secondly, data are in agreement with previous studies showing enhanced levels of brain-infiltrating T cells but not elevated numbers of B cells in the early stage of CDV infection of dogs^9^, and, thirdly, detected demyelinating lesions lacked high-grade concomitant inflammation. Based on findings in other studies, inflammatory cell immigration increases substantially only in late subacute to chronic demyelinating plaques of canine distemper, whereas early lesions are rather characterized by white matter vacuolization and frank demyelination^47,68^. Moreover, the presence of glial cells within lesions in the present study matches the descriptions for early demyelinating disease in CDV-infected dogs^9^. Axonal damage was present in all brain regions in the present study^69^, which also supports the conclusions that CDV-infected foxes suffered from an early-stage CNS disease, given that axon pathology has been shown to precede myelin loss in infectious disorders^70,71^.

Amino acid variation in the receptor binding domain of the CDV H protein has been previously proposed to have a role in optimizing use of heterologous cellular receptors during cross-species virus transmission to non-canid host species^34,72^. The CDV strains present in infected foxes belong to three closely related subclades of the Europe-1/South America-1 genotype, with three strains from the Germany-IV subclade displaying a Y549H mutation in the SLAM binding region of the H protein. Previous studies have suggested that this particular residue may show a bias towards histidine in wildlife tissues as compared to a tyrosine in canid tissues^7,34,61,72–77^. Thus, the Y549H change observed in three foxes, a canid species, is suggestive of transmission from other wildlife such as raccoons, martens or badgers. However, additional factors must also have a role in host determination given that previous studies have also reported canid-derived viruses with the 549H mutation^34^.

Interestingly, although there are clear differences with respect to nucleotide and amino acid homology, the CNS disease manifestation induced by the different CDV strains did not seem to differ, i.e., no patterns in the neuropathology related to sub-lineage affiliation could be observed in the present study. However, further analysis of pathological changes described in animals infected with virus strains closely related to the strains identified in this study may help to identify strain-specific pathologic features. A strain of CDV from Hungary, which is closely related to the fox CDV strains in the Germany-II, -III, -IV subclades caused neurological signs associated with lymphoplasmacytic encephalitis in dogs^78^. Other closely related Europe-1 genotype strains are also associated with neurologic signs in the affected animals^33,39,79^ with polioencephalitis more commonly observed than demyelination^39^. CDV strains present in two foxes with neurological symptoms in Germany in 2008 are closely related to the one fox strain (288/17) in the present study but limited data is available from these cases^34^. Similarly, no information is available concerning the pathology induced by another closely related CDV strain following an outbreak on a Danish mink farm^61^.

The finding that the virus derived from animal 288/17 had an additional N-linked glycosylation site at aa position 584 is interesting since this seems to be a feature limited to viruses belonging to the Asia-1 genotype^80,81^. N-linked glycosylation plays a role in determining the antigenicity of many proteins and can modulate virus entry capacity or immune evasion^82–84^. Viral proteins are glycosylated in the endoplasmic reticulum of the host cell, requiring an N-X-S/T (asparagine—any aa apart from proline—serine or threonine) aa sequence and a stable conformation of the protein with accessibility of the site to be glycosylated. However, the exact function of N-linked glycosylation in morbillivirus H protein apart from keeping it in the right conformation has not been fully determined yet^84^.

Although strain-to-strain variation might have a role in determining the disease phenotype, further interpretations are confounded by animal-to-animal variation and the comparatively small sample size of eighteen foxes in our study. Heterogeneity with respect to age, sex, immunological status and unknown time from infection to death, can all influence the disease course^9,51,85–87^.

Analysis of CDV cell tropism via immunofluorescence dual labelling showed that Iba1^+^ microglia/macrophages was the predominant CDV-infected cell type in the cerebrum, cerebellum and brainstem of foxes. In other studies in dogs und foxes, neurons and/or astrocytes have been reported to be the primary target cells for CDV infection in the CNS^36,37,39,85,88,89^. Unfortunately, many studies on CDV-infected foxes do not differentiate between different types of glial cells. Some authors describe only limited infection of glial cells^35,39^, while others report these to be the main target cells^37,40^. Interestingly, a study on strains closely related to the sub-lineage 2 strains reported only a small number of infected glial cells, even though other findings were complementary to the present study^39^. Ex-vivo flow cytometry analysis of microglia in dogs with severe demyelination yielded only 18% of CDV-positive microglia^55^. The question thus arises as to whether the high levels of CDV antigen identified within microglia/macrophages in the present study originates from phagocytosed material from other cell types killed by CDV infection, rather than from direct infection. Indeed, phagocytotic activity was found to be enhanced in CDV-infected microglia in dogs^55^. Microglia are the main effector cell type of the immune system in the brain^90^, and therefore undergo profound changes upon CDV infection^55^. In the present study, accumulation of Iba1^+^ microglia/macrophages was associated with an enhanced expression of MHC II. MHC II is upregulated upon various viral infections^91^, including CDV infection of dogs^55,92^. Microglia have been associated with early demyelination in canine distemper as they proliferate^93^, present antigen, activate T cells and release reactive oxygen species^55^ which collectively cause inflammation and CNS damage. Similar to CDV-infected dogs, only limited infection of oligodendrocytes was apparent in brains of infected foxes^53,94^.

The low amount of CDV N protein in the present study confirms previous data showing the presence of viral RNA but restricted protein translation in CDV-infected dogs^95^. A lack of correlation between CDV antigen and RNA levels has been reported previously in dogs with distemper^96^, with differences mainly in gray matter areas. An additional finding in the present study was that only a maximum of 59% of foxes showed CDV N antigen expression in one specific brain region, while almost all foxes showed CDV mRNA in all brain regions^9,46,48,96^.

Apart from microglia, pro-inflammatory cytokines and myelinotoxic factors released by astrocytes might contribute to early demyelination^67,97^. Previous studies have reported astrocytes to be the main target cells of CDV infection in dogs; however, in the present studies they constituted the third-most infected cell type after microglia/macrophages and neurons. Nesseler et al.^48^ report neurons and protoplasmic astrocytes to be the main target cells also in CDV inclusion body polioencephalitis of dogs. Besides being viral targets, astrocytes participate in the formation of the blood–brain-barrier^98^. Upon CDV infection, astrocytes react by hypertrophy, hyperplasia and change of their protein expression profile^52,89,99–101^. In the present study, an upregulation of GFAP was found in the cerebral gray matter and brainstem, indicating astrocytic activation^57,102^. This matches the findings in regard of histologic lesion distribution, where the cerebral gray matter and brainstem of foxes were identified to be affected most commonly and most severely. Interestingly, reduced GFAP staining intensities and an increase of vimentin^+^ astrocytes were observed within the center of foci containing abundant CDV antigen. Similarly, an increase of vimentin^+^ immature astrocytes can be observed in CDV-induced brain lesions of dogs, representing a potential target for the virus^103–105^. Reversion to vimentin expression and to an immature phenotype of astrocytes represents a reactive change, which has been observed also in human neuropathologic conditions such as multiple sclerosis and Alzheimer’s disease^103,106–109^.

In the present study, the brainstem, cerebral gray matter and cerebellar white matter seemed to be the most affected areas. In these regions, most apoptotic cells were present, too. Cerebral gray matter and brainstem were the regions where most foxes showed lesions and the severity of these lesions was the highest, whereas the brainstem and cerebellar white matter were most affected by demyelination. Moro et al.^110^ reported apoptosis of granular cells in the cerebellar gray matter of CDV-infected dogs. These authors suggested a “spillover” of infection into these cells from the neighboring white matter areas, which led to apoptosis of the neurons. Subsequent neuronal cytolysis has been proposed to contribute to the demyelination process^54,111^. Yet only few animals with demyelinating lesions and comparatively few apoptotic cells were found in the present survey, so apoptosis seems to play a minor role for lesion development in CDV-infected foxes.

In the course of the ongoing epidemic of canine distemper over the last fifteen years, foxes have been one of the most affected species^62^. This undoubtedly facilitates cross-species virus transmission, because the roaming behavior of foxes including proximity to human settlements has been associated with the spread canine distemper^29,32,112,113^. Foxes can contract CDV via contact with unvaccinated dogs or alternatively transmit the virus to other susceptible species, thus serving as an initial source of CDV outbreaks wildlife infection^114^. This poses a serious threat to captive and free-ranging endangered wildlife species^115–118^, especially given sinking vaccination rates in dogs and diminished vaccine efficiencies in some species^115–119^. In summary, our study contributes to a better understanding of the pathologic processes in foxes evoked by CDV, which is important in the scope of understanding the impact of this virus infection upon wild animals.

## Materials and methods

### Samples

Organ material of 18 foxes was included in this study, of which 13 originate from the region of Arnsberg in North-Rhine Westphalia and five from Lower Saxony (Figure S1). Foxes were either killed by hunters due to abnormal behavior (n = 15) or found dead (n = 3). Necropsies were performed and tissue samples (brain, lung, spleen, liver, kidney, adrenal gland, bladder, trachea, lymph node, tonsil and heart) were stored in 10% formalin before embedding in paraffin. Histopathological and immunohistochemical analysis of CDV nucleocapsid (N) protein distribution was performed on tissues sections of cerebrum, cerebellum and brain stem from the CNS and lung, spleen, bladder, kidney, liver, and intestine. Of one fox only brainstem material was available for analysis. Serial sections of 3 µm were cut and mounted on SuperFrost-Plus (Menzel Gläser, Braunschweig, Germany) microscope slides. Negative controls consisted of tissue sections of brain samples from four foxes, which had been submitted for necropsy from 2017 to 2018 and were shown to be negative for CDV infection by immunohistochemical staining.

### Immunohistochemistry and in-situ-hybridization

Immunohistochemistry used for the detection of CDV N protein, Iba1, MHC II, GFAP, vimentin, CD3, Pax5, CNPase, β-APP and cleaved caspase-3 were performed as described^120^. Briefly, sections were de-waxed in Roticlear® (Carl Roth, Karlsruhe, Germany) and consecutively re-hydrated in alcohols with endogenous peroxidase inhibited by immersing sections in 85% ethanol. Sections were then incubated overnight with the indicated antibodies (Supplemental Table S2). For negative controls, ascites fluid from non-immunized -BALB/c mice or serum from non-immunized rabbits was applied instead of the respective antibody. Visualization of positive antigen–antibody reaction was achieved by incubation with 3,3′-diaminobenzidine tetrahydrochloride (DAB) with 0.03% H2O2, pH 7.2 for 5 min, followed by slight counterstaining with Mayer’s hemalaun. Details of antibodies used are given in Supplemental Table S2.

In situ hybridization was performed as described before^94^. Digoxigenin (DIG)-labeled CDV-specific probes against N mRNA were used, which had been generated from cDNA by SP6/T7 transcription run off method (RiboMAX®-system, Promega Corporation, Madison, USA). Sections were dewaxed in a series of washes in xylene, hydrated with graded ethanol and lastly washed in ultrapure H2O. Proteolytic digestion, postfixation, acetylation and prehybridization were performed, followed by actual hybridization overnight at 52 °C in a moist chamber, at a probe concentration of 100 ng/ml. Finally, visualization was achieved by a 1:200 dilution of anti-DIG antibody which was conjugated with alkaline phosphate (Merck, Germany) which was followed by the addition of substrates nitroblue tetrazoliumchloride (NBT) and 5-bromo-4-chloro-2-indolyl phosphate (BCIP, X-Phosphate). A pellet of DH82 cells persistently infected with the CDV Onderstepoort strain fixed with formalin and embedded in paraffin served as positive control. The negative control consisted of a pellet of the same cells, which was only treated with the hybridization buffer without probe.

### Immunofluorescence

Sections were de-waxed by sequentially submerging in RotiClear® (Carl Roth, Karlsruhe, Germany) (twice), 100% isopropanol and 100% ethanol (5 min each), washed in phosphate-buffered saline (PBS, pH 7.0) for 3 × 5 min on a magnetic stirrer, followed by boiling in citrate buffer in a microwave at 800 W for 20 min. Sections were cooled at room temperature (RT) for 10 min, prior to another washing step for 2 × 5 min in PBS. Subsequently, slides were transferred to CoverPlates in Shandon Sequenza® immunostaining chambers (Thermo Fisher Scientific, Waltham, USA) and overlaid with PBS. Blocking solution consisting of PBS + 0,1% 2-[4-(2,4,4-trimethylpentan-2-yl)phenoxy]ethanol (Triton X-100) + 1% bovine serum albumin (BSA) + 25% goat normal serum was applied at 150 µL per section and incubated for 30 min at RT. Primary antibodies were diluted in PBS + 0,1% Triton X-100 + 1% BSA. CDV D110 was used at a 1:100 dilution and mixed with a polyclonal antibody specific for neurons (NeuN), astrocytes (GFAP) and microglia/macrophages (Iba1), respectively (Supplemental Table S3). For CDV/CNPase dual labeling, a mouse monoclonal anti-CNPase antibody was used in combination with a rabbit polyclonal anti-CDV-N antibody. BALB/c mouse serum (1:1000 dilution) and normal rabbit serum (1:3000) diluted in PBS + 1% BSA were mixed in equal ratios and served as a negative control. 150 µL of diluted primary antibodies or control serum were applied to each section and incubated at 4 °C overnight. Unbound antibody was removed by three washes for 5 min in PBS before applying 150 µL of secondary antibody solution which consisted of 1:200 dilutions of goat anti-mouse Alexa Fluor 488 (115–545-003 G pAb, Jackson ImmunoResearch, West Grove, USA) or goat anti-rabbit Cy3 (G pAb 111-165-144, Jackson ImmunoResearch, West Grove, USA) prepared in PBS + 0.1% Triton X-100 + 1% BSA. After 45 min of incubation at RT in the dark 3 × 5 min PBS washes were performed, followed by counterstaining of cell nuclei by the addition of 150 µL/section bisbenzimide (1% in sterile ddH2O) for 5 min. Subsequently, slides were washed with dH2O twice and covered with Dako Fluorescence Mounting Medium® (Agilent Technologies, Santa Clara, USA) and a glass coverslip. Samples were stored at 4 °C in the dark and dried overnight before microscopical assessment using a Zeiss fluorescence microscope Axio Imager M2. Total numbers of CDV-positive cells were counted, and the percentage of double-reactive cells was determined for each cellular marker.

### Analysis of slides

To assess the lesion types present in the fox brains, H&E-stained sections of the brainstem, cerebellum and cerebrum were analyzed and lesion types occurring in the brainstem, cerebellar gray matter, cerebellar white matter, cerebral gray matter, cerebral white matter and hippocampus were noted for each animal.

For the analysis of immunohistochemistry and in situ hybridization, slides were digitalized and area of brainstem, cerebellum and cerebrum was measured using the microscope BZ-9000 BIOREVO with the BZ-II Analyzer software (Keyence Corporation, Osaka, Japan). The size of different brain areas (mm^2^) was assessed by manually circumscribing them with the area measurement tool of the software^121^. For CDV N antigen, CDV ISH, β-APP and cleaved caspase-3, all positive cells or axons were counted per brain region (cerebral gray matter, cerebral white matter, hippocampus, cerebellar gray matter, cerebellar white matter and brainstem). The counted numbers were set in relation to the size of the area and data expressed as mean cells per mm2 and labeled axons per mm2, respectively. Similarly, for myelin loss (LFB and anti-CNPase staining), the number of foci which showed a pallor in the respective staining was counted per brain region and related to the respective area size, with data expressed as mean number of foci/mm^2^.

For immunofluorescence dual labeling, for each CDV/marker combination in each slide (containing either cerebrum or cerebellum with brainstem) the total number of CDV+ cells was counted, followed by the number of cells expressing both CDV and the cell-specific marker. Subsequently, the number of CDV/marker double-positive cells was set in relation to the total number of CDV+ cells for each slide per animal.

The number of Iba1+, MHC II+, GFAP+, vimentin+, CD3+ and Pax5+ cells were analyzed semi-quantitatively in each brain region: (score 0 = no immunoreactivity; 1 = single foci/cells, < 5%; 2 = multifocal reactivity, 5–30%; 3 = multifocal to diffuse reactivity, > 30% of brain region stained.

### Statistical analyses

GraphPad Prism 9 software was used for all statistical analyses. Group comparison between the brain areas for CDV N antigen, CDV RNA, β-APP, cleaved caspase-3, LFB and CNPase were performed by one-way ANOVA comparing the means of each group to the means of every other group, correcting for multiple comparisons by Tukey’s test for multiple comparisons and Šídák's multiple comparisons test. Immunofluorescence dual labeling was analyzed by comparing the mean ratio of cells double-positive for CDV and the respective cellular marker to total CDV^+^ cells in both brain regions (cerebrum and cerebellum with brainstem) between each CDV/marker combination in the respective brain area (cerebrum or cerebellum with brainstem) by a mixed-effects analysis. For the Iba1, MHC II, GFAP, vimentin, CD3 and Pax5 immunohistochemistry, mean scores were compared to control foxes by using the Mann–Whitney-U test. Generally, a p value ≤ 0.05 was considered significant. All tests were performed as two-tailed tests. Significance levels were indicated by asterisks in the graphs, with the following significance levels: *: p ≤ 0.05, **: p ≤ 0.01, ***: p ≤ 0.001, ****: p ≤ 0.0001.

### RT-PCR and phylogenetic analyses

RNA was extracted from frozen brain tissue obtained from the brains of 13 foxes using the QIAamp viral RNA mini kit (QIAGEN). Reverse transcription to obtain cDNA was performed with SuperScript IV Reverse Transcriptase (Thermo Fisher Scientific) according to the recommended protocol. The CDV H gene ORF was amplified by PCR using primers homologous to Europe 1 strains (Supplemental Table S4) with Q5 High-Fidelity DNA polymerase (NEB) following the standard protocol. The resulting amplicons were purified using the Monarch Gel Extraction kit (NEB) and Sanger sequenced (Microsynth Seqlab) using Europe-I specific primers (Supplemental Table S4). The resulting sequences were assembled to complete consensus CDV H open reading frames and aligned with 600 CDV H ORFs available from GenBank. The best model for phylogenetic analysis was chosen by the MEGA X software as the General Time Reversible model with a Gamma distribution.

A maximum-likelihood phylogenetic tree with 1000 bootstrap values was generated using the same software. Comparative analysis of selected H amino acid sequences was performed using the Clustal omega tool (https://www.ebi.ac.uk/Tools/msa/clustalo/) and MegAlign software (DNASTAR, Lasergene version 12, Madison, WI, USA). The H gene sequences obtained from 10 samples are available at GenBank under the following accession numbers: OL795421, OL795422, OL795423, OL795424, OL795425, OL795426, OL795427, OL795428, OL795429 and OL795430.

Glycosylation sites were predicted by uploading the protein sequences of each H protein to the DTU Health Tech NetNGlyc-1.0 webserver (https://services.healthtech.dtu.dk/service.php?NetNGlyc-1.0).

### Institutional review board statement

Clinical samples from the CDV-infected fox cases reported in this study were collected by the Chemisches und Veterinäruntersuchungsamt in Westfalen for the purpose of viral diagnostics. Frozen tissue samples were transported to the Research Center for Emerging Infections and Zoonoses, University of Veterinary Medicine Hannover for specific genotyping of CDV strains under permit number DE 03 201 0043 21 obtained from the Fachbereich Öffentliche Ordnung, Gewerbe- und Veterinärangelegenheiten, Landeshauptstadt Hannover.

## Supplementary Information

Supplementary Information.

## Data Availability

Hemagglutinin sequences were deposited on GenBank (https://www.ncbi.nlm.nih.gov/genbank/) under the accession numbers mentioned in the materials & methods section. All other data generated or analysed during this study are included in this published article (and its Supplementary Information files).
