# Supplementary material for: Neuropathologic and molecular aspects of a canine distemper epizootic in red foxes in Germany
Source: Sci Rep. 2022 Aug 29;12:14691. doi: 10.1038/s41598-022-19023-9 (PMC9424316; doi:10.1038/s41598-022-19023-9)
Supplement: Supplementary file 1 — Supplementary Information. [file 41598_2022_19023_MOESM1_ESM.pdf]

Neuropathologic and molecular aspects of a canine distemper epizootic in red foxes in Germany

Supplementary material

Figure S1

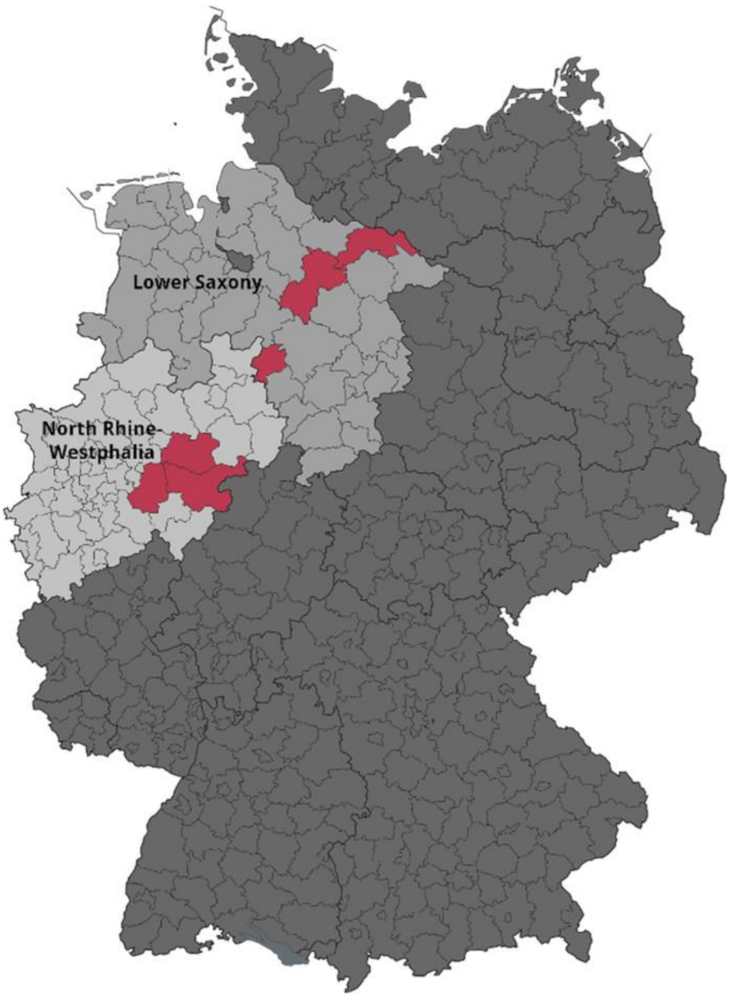

Figure S1. Map of Germany showing the geographical origin of fox samples used in this study. The states of Lower Saxony and North Rhine-Westphalia are shaded in light grey, the administrative districts where the foxes were found or shot are highlighted in red. Image modified from " Landkreise, Kreise und kreisfreie Städte in Deutschland 2007-07-01 - 2008-07-31" by M. Stadthaus, 2007, Wikimedia Commons, URL: https://commons.wikimedia.org/wiki/File:Landkreise,_Kreise_und_kreisfreie_St%C3%A4dte_in_Deutschland_2007-07-01_-_2008-07-31.png[1], access date: 05/11/2021.

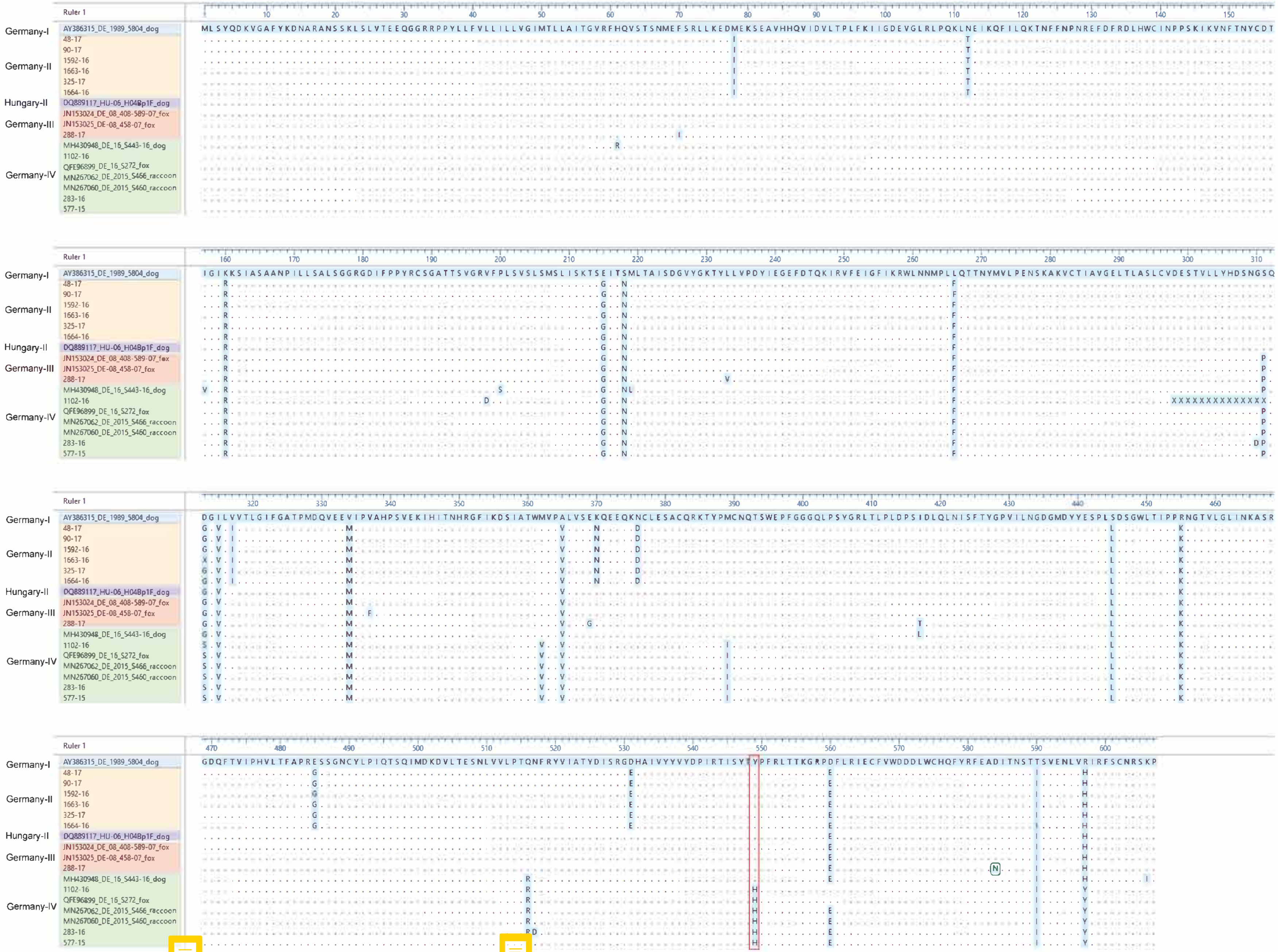

Figure S2. Tabular overview of amino acid changes in the Hemagglutinin protein of foxes from this study (48-17, 90-17. 1592-16, 1663-16, 325-17, 1664-16, 288-17, 1102-16, 283-16, 577-15) in comparison to closely related strains. The different sub-clades are highlighted in color. Variation in amino acid 549 (red box) has been associated with either wildlife or canid infections.The highlighted aa change at position 584 leads to an additional N-linked glycosylation site. An X indicates missing sequence information for this position.

Table S1. Additional sample data including CDV antigen amounts determined by immunohistochemistry and presence of further pathogens.

Year of death	animal	state	sex	age	weight	cause of death	distemper IHC	other pathogens
2015	577/15	NRW	male	adult	n.d.	killed	brain: +<br>lung: +<br>trachea: +<br>lymph nodes: +<br>spleen: +<br>liver: +<br>heart: -	
2016	283/16	NRW	female	adult	4.2 kg	killed	brain: +<br>lung: +<br>spleen: +<br>urinary bladder: +<br>kidney: +<br>liver: +<br>stomach: +<br>heart: -	Liver: *Staphylococcus spp.*
2016	1663/16	NRW	male	juvenile	4 kg	killed	brain: +<br>lung: +<br>trachea: +<br>lymph node:+<br>spleen: +<br>urinary bladder: +<br>kidney: +	
2016	1592/16	NRW	female	adult	5.4 kg	killed	brain: +<br>lung: -<br>lymph node: -<br>spleen: +<br>urinary bladder: -<br>kidney: -<br>liver: -<br>stomach: -	
2016	1102/16	NRW	male	adult	6 kg	killed	brain: +<br>lung: +<br>lymph nodes: +<br>spleen: -<br>urinary bladder: +<br>liver: -<br>heart: -	
2016	1664/16	NRW	female	juvenile	3.8 kg	killed	brain: +<br>lung: +<br>spleen: -<br>urinary bladder: +<br>liver: -<br>stomach: +<br>heart: -	
2017	325/17	NRW	male	adult	5.6 kg	killed	brain: +<br>trachea: +<br>stomach: +<br>urinary bladder: +<br>tonsil: +	
2017	288/17	NRW	male	n.d.	4.2 kg	killed	brain: +<br>lung: +<br>trachea: +<br>lymph nodes: +<br>tonsil: +<br>spleen: -<br>urinary bladder: +<br>liver: -<br>stomach: +<br>heart: -	

Table S1 (continued)

Year of death	animal	state	sex	age	weight	cause of death	distemper IHC	other pathogens
2017	100/17	NRW	female	adult	3.4 kg	killed	brain: +<br>lung: -<br>tonsil: -<br>spleen: -<br>kidney: -<br>liver: -<br>adrenal gland: -<br>stomach: -<br>intestine: -	
2017	276/17	NRW	male	adult	6 kg	killed	brain: +<br>lung: +<br>trachea: +<br>lymph node: -<br>tonsil: +<br>salivary gland: -<br>spleen: -<br>urinary bladder: +<br>kidney: -<br>adrenal gland: -<br>liver: -<br>stomach: -<br>heart: -	Forelimb:<br>*Streptococcus suis*
2017	90/17	NRW	male	juvenile	5 kg	killed	brain: +<br>lung: -<br>trachea: -<br>lymph node: -<br>spleen: -<br>urinary bladder: -<br>kidney: -<br>adrenal gland: -<br>liver: -<br>stomach: -<br>heart: -	
2017	48/17	NRW	female	adult	4.2 kg	killed	brain: +<br>lung: -<br>trachea: -<br>spleen: -<br>urinary bladder: -<br>kidney: -<br>liver: -<br>heart: -	
2017	313/17	NRW	male	adult	4.4 kg	killed	brain: +<br>lung: +<br>trachea: -<br>tonsil: -<br>spleen: +<br>urinary bladder: +<br>kidney: -<br>adrenal gland: -<br>liver: -<br>stomach: -<br>heart: -	Spleen and kidney:<br>*Streptococcus canis,*
2017	510-17-232	LS	male	adult	6.2 kg	died	brain: +	*Enterobacter amnigenus*
2017	510-17-625	LS	n.d.	n.d.	n.d.	killed	brain: +	
2017	510-17-320	LS	female	adult	4.7 kg	died	brain: +	
2017	510-17-954	LS	male	juvenile	3.2 kg	killed	brain: +	
2018	510-18-356	LS	male	adult	8.0 kg	died	brain: +	

n.d.: not determined, +: positive, -: negative, NRW: North-Rhine Westphalia, LS: Lower Saxony.

Table S2. Details of antibodies used for immunohistochemistry.

Antibody	Target	Antigen	Dilution factor	Origin
D110, Ms mAb	CDV	N protein	1:1000	kindly provided by Prof. Dr. A. Zurbriggen, University of Bern, Switzerland
DakoCytomation Z0334, Rb pAb	astrocytes	GFAP	1:2000	Agilent, Santa Clara, USA
DakoCytomation M0725 V9, Ms mAb	immature astrocytes	Vimentin	1:100	Agilent, Santa Clara, USA
Wako Chemicals, 019-19741, Rb pAb	Microglia/ macrophages	Iba1	1:500	Fujifilm Wako Pure Chemical Corporation, Osaka, Japan
Dako Cytomation M0746, Ms mAb	antigen-presenting cells	MHC II	1:100	Agilent, Santa Clara, USA
Dako Cytomation A0452, Rb pAb	T cells	CD3	1:500	Agilent, Santa Clara, USA
BD Biosciences, 610862 clone 24, Ms mAb	B cells	Pax5	1:100	BD Biosciences Pharmingen, San Diego, USA
Merck/Millipore Chemicon CNPase mono MAB 326, Ms mAb	myelin/oligoden-drocytes	CNPase	1:100	Merck, Darmstadt, Germany
Cell Signaling Technology, #9661, Rb pAb	apoptotic cells	Cleaved caspase-3 (Asp 175)	1:200	Cell Signaling Technology, Danvers, USA
Merck/Millipore MAB348 22C11, Ms mAb	axonal damage	β-APP	1:800	Merck, Darmstadt, Germany

N: nucleoprotein, GFAP: glial fibrillary acidic protein, Iba1: ionized calcium binding adaptor molecule 1, MHC II: major histocompatibility complex II, CD3: cluster of differentiation 3, Pax5: paired-box 5, CNPase: 2',3'-cyclic-nucleotide 3'-phosphodiesterase, Asp: aspartic acid, β-APP: beta-amyloid precursor protein, ms: mouse, rb: rabbit, mAb: monoclonal antibody, pAb: polyclonal antibody

Table S3. Details of antibodies used for immunofluorescence staining

Antibody	Target	Antigen	Dilution factor	Origin
D110, Ms mAb	CDV	N protein	1:100	kindly provided by Prof. Dr. A. Zurbriggen, University of Bern, CH
Anti-CDV-NP (#25), Rb pAb	CDV	N protein	1:2000	Kindly provided by Dr. C. Örvell, PhD, The National Bacteriological Laboratory, Stockholm, S
Abcam, ab 177485, Rb mAb	neurons	NeuN	1:200	Abcam plc, Cambridge, UK
DakoCytomation Z0334, Rb pAb	astrocytes	GFAP	1:500	Agilent, Santa Clara, USA
Wako Chemicals, 019-19741, Rb pAb	microglia/ macrophages	Iba1	1:250	Fujifilm Wako Pure Chemical Corporation, Osaka, Japan
Merck/Millipore Chemicon CNPase mono MAB 326, Ms mAb	myelin/oligo-dendrocytes	CNPase	1:100	Merck, Darmstadt, Germany

N: nucleoprotein, NeuN: neuronal nuclei, GFAP: glial fibrillary acidic protein, Iba1: ionized calcium binding adaptor molecule 1, CNPase: 2',3'-cyclic-nucleotide 3'-phosphodiesterase

Table S4. Primer sequences used to amplify and sequence fox H genes.

Primer name	Sequence 5'-3'	Function	Reference
CDV_7059f	agggctcaggtagtccaaca	Amplification of CDV H open reading frame	This study
CDV_9022r	aggatcctggatcctyagtttttttataatg		
CDV_6926f	tactctgatcacacgtctta	Sanger sequencing	Sekulin et al. (2011)[2]
CDV_7652 r	tagctccactgcatctgtat		
CDV_7326 f	ccgtacatcaccaagtcata		
CDV_8026 r	tagaacaccaccttgtgaac		
CDV_7967 f	gtagatgagagcaccgtatt		
CDV_8625 r	tgtgtaggcaacaccact		
CDV_8483 f	ggagaccagttcactgtaat		
CDV_8483 f	gatggacctcaggatataga		

H: hemagglutinin, f:forward, r: reverse

Supplemental references

1 Stadthaus, M. (https://commons.wikimedia.org/, 2007).

2 Sekulin, K. *et al.* Emergence of canine distemper in Bavarian wildlife associated with a specific amino acid exchange in the haemagglutinin protein. *Vet J* **187**, 399-401, doi:10.1016/j.tvjl.2009.12.029 (2011).
